# Chaperonin-Containing t-Complex Protein-1 Subunit ***β*** as a Possible Biomarker for the Phase of Glomerular Hyperfiltration of Diabetic Nephropathy

**DOI:** 10.1155/2015/548101

**Published:** 2015-04-05

**Authors:** Chung-Ze Wu, Li-Chien Chang, Yuh-Feng Lin, Yi-Jen Hung, Dee Pei, Jin-Shuen Chen

**Affiliations:** ^1^Graduate Institute of Clinical Medicine, College of Medicine, Taipei Medical University, Taipei, Taiwan; ^2^Division of Endocrinology and Metabolism, Department of Internal Medicine, Shuang Ho Hospital, Taipei Medical University, Taipei, Taiwan; ^3^School of Pharmacy, National Defense Medical Center, Taipei, Taiwan; ^4^Division of Nephrology, Department of Internal Medicine, Shuang Ho Hospital, Taipei Medical University, Taipei, Taiwan; ^5^Division of Endocrinology and Metabolism, Department of Internal Medicine, Tri-Service General Hospital, National Defense Medical Center, Taipei, Taiwan; ^6^Division of Endocrinology and Metabolism, Department of Internal Medicine, Cardinal Tien Hospital, Medical School, Catholic Fu Jen University, Xindian, Taiwan; ^7^Division of Nephrology, Department of Internal Medicine, Tri-Service General Hospital, No. 325, Section 2, Cheng-Kung Road, Neihu, Taipei 114, Taiwan

## Abstract

In cell model, we discovered the association between chaperonin-containing t-complex polypeptide 1 subunit *β* (TCP-1*β*) and early diabetic nephropathy (DN). In this study, we further explored the relationships between TCP-1*β* and type 2 diabetic mellitus (DM). To mimic the clinical hyperfiltration state, a type 2 DM mice model was established by feeding a high-fat diet in combination with treatment of streptozotocin and nicotinamide. Blood and urine were collected to determine creatinine clearance (*C*_cr_), and kidney tissues were harvested for evaluation of TCP-1*β* expression by immunohistochemistry and Western blot. Meanwhile, clinical subjects of healthy controls and type 2 DM were recruited to strengthen the evidence with urine TCP-1*β*. Results showed that *C*_cr_ and the expression of TCP-1*β* in kidney were significantly higher one week after hyperglycemia development, suggesting that the hyperfiltration state was successfully established in the mice model. TCP-1*β* was expressed predominantly on renal tubules. By using the estimated glomerular filtration rate to index progression in clinical investigation, urine TCP-1*β* level was associated with the hyperfiltration phase in type 2 DM patients. Conclusively, we confirmed that TCP-1*β* is a possible biomarker for early nephropathy of type 2 DM, but further mechanistic study to elucidate its cause and pathway is needed.

## 1. Introduction

Diabetic mellitus (DM), in particular types 1 and 2, is a global epidemic, contributing to a huge economic burden for health care around the world [1, 2]. DM leads to many complications, such as cardiovascular disease, nephropathy, retinopathy, and neuropathy. While diabetic nephropathy (DN) is a leading cause of end-stage renal disease (ESRD), both DM types have a certain percentage of subjects developing DN. For the progression of type 1 DN, there are five predictable stages: glomerular hyperfiltration, silent, microalbuminuria, macroalbuminuria, and ESRD. As for the progression of type 2 DN, it may show a similar phenotype as type 1 DN from the early stage of glomerular hyperfiltration to ESRD. The prevalence of glomerular hyperfiltration in types 1 and 2 DN is 25–75% and 5–40%, respectively [3]. Although not all the progression of DN begins from glomerular hyperfiltration, the appearance of glomerular hyperfiltration has been suggested to correlate with the development of ESRD [4]. Given the deleterious effect of glomerular hyperfiltration on the progression of DN, understanding the mechanism of glomerular hyperfiltration is important to prevent further renal deterioration.

There is no consensus on the definition of glomerular hyperfiltration. It is generally defined by a glomerular filtration rate (GFR) of more than 120 mL/min, since the glomerular and tubular hyperfiltration theories in DN have been mainly based on experiments in rodent models [3]. While hyperfiltration is caused by increased intraglomerular capillary pressure, which may be modulated by afferent and efferent arteriolar, the permeability of the capillary membrane, and the difference between the hydraulic and oncotic pressure gradients, one mechanism noted in glomerular theory is mesangial cell hypocontraction, which leads to a decrease in capillary surface area and capillary permeability, therefore accentuating the GFR [5, 6]. In our previous studies, an* in vitro* high glucose-induced mesangial cell hypocontractility model was established to explore the underlying mechanism leading to glomerular hyperfiltration. We suggest that chaperonin-containing t-complex polypeptide 1 subunit *β* (TCP-1*β*) may play an important role in mesangial cell hypocontractility in the development of DN. However, the role and expression of TCP-1*β in vivo* in early DN have not been discussed until this study.

In this study, we established a type 2 DM mice model to mimic the progression of DN and evaluated the expression of TCP-1*β* in the kidney. Meanwhile, we investigated urine TCP-1*β* levels in the clinical subjects with type 2 DM at the glomerular hyperfiltration stage.

## 2. Material and Methods

### 2.1. Induction of Type 2 DM Mice Model with Glomerular Hyperfiltration

Streptozotocin (STZ) (N-nitroso derivative of glucosamine, Sigma, S0130) is a broad-spectrum antibiotic extracted from streptomyces achromogenes. It is a pancreatic beta-cell toxin that induces rapid and irreversible necrosis of *β* cells and is widely used in making experimental DM mouse models. In our study, male BALB/c mice were obtained from the National Laboratory Animal Breeding and Research Center (Taipei, Taiwan). For induction of type 2 DM, mice were housed in laboratory cages and fed with a high-fat (HF) diet (40% fat, Research Diets, Inc., NJ) [7] for 3 weeks. Subsequently, mice received 75 mg/kg and 150 mg/kg of intravenous STZ, 5 days apart. Nicotinamide (NTM) (1.5 g/kg, dissolved in saline) was injected intraperitoneally 15 minutes (mins) before each injection of STZ. All animals were nonfasted at the time of STZ administration. After induction, blood glucose was measured daily by tail-vein sampling using a glucometer (Roche, ACCUCHEK). Animals with blood glucose more than 11.1 mmol/L (200 mg/dL) were included in this study. After development of type 2 DM, murine urine and blood were collected at intervals of 1 week. At each interval, at least 6 mice were sampled. In addition, kidney tissues were harvested after mice were euthanized in the first and fourth weeks. During induction, at given times, GFR was estimated by a *C*_cr_ equation. *C*_cr_ in milliliters of plasma per minute was calculated by *C*_cr_ = (*C*_*u*_/*C*_*p*_) × *V*/body weight (g), where *C*_*u*_ is the concentration of creatinine in urine, *C*_*p*_ is the concentration of creatinine in plasma, and *V* is the urine flow rate in milliliters per minute [8]. For verifying the establishment of type 2 DM, the homeostasis model assessment-insulin resistance (HOMA-IR) in mice was also evaluated by HOMA-IR = insulin (*μ*U/mL) × glucose (mmol/L)/22.5 [9]. Blood and urine creatinine levels were determined according to previous methodologies [10]. The experimental animal protocol was approved by the Institutional Animal Care and Use Committee at the National Defense Medical Center, Taipei, Taiwan (approval number: IACUC12-058).

### 2.2. Immunohistochemical (IHC) Staining

The kidney tissue was fixed in 10% formaldehyde fixative solution and embedded in paraffin. Sections of formalin-fixed kidney tissue were immersed in xylene for 5 mins three times to remove paraffin, followed by incubation with phosphate-buffered saline (PBS) and 1% bovine serum albumin (BSA) at room temperature (RT) for 30 mins for blocking. After removing paraffin and rehydrating, the slices were incubated with 1 : 50 dilution of the primary antibody, rabbit anti-TCP-1*β* antibody (Santa Cruz, sc-28556, CA) in PBS at 4°C overnight. Subsequently, the slices were incubated with 1 : 50 dilution of secondary antibody, biotinylated anti-rabbit antibody (Vector laboratories, BA-1300, CA), for 40 mins, and washed with Tris-buffered saline containing 0.05% Tween 20 (TBST; pH 7.4). The sections were then treated with VECTASTAIN ABC (Vector laboratories, CA) working solution for 30 mins. The reaction was visualized by use of BAD chromogen. The slices were then observed with an optical photomicroscope. Negative controls were performed by omitting primary antibodies.

### 2.3. Western Blot (WB)

Equal amounts of renal cortex protein (30 *μ*g) from each sample were separated by 8% SDS-PAGE gel. The gel was electroblotted onto a nitrocellulose membrane, incubated for 1 hour in blocking buffer (TBST, 2% bovine serum albumin), washed three times in TBST, and incubated with 1 : 500 dilutions of TCP-1*β* in TBST at 4°C overnight. Blots were washed three times and incubated in horseradish peroxidase-conjugated anti-rabbit antibody for 1 hour at RT. Membranes were washed three times, and the membrane-bound antibody detected was incubated with Western blot detection system and captured on X-ray film.

### 2.4. Clinical Subjects and Study Design

The subjects in this study were enrolled in Tri-Service General Hospital and were divided into three groups: control, DM with hyperfiltration (estimated GFR; eGFR > 120 mL/min), and DM with nonhyperfiltration (eGFR < 120 mL/min). All of 17 healthy subjects and 148 patients with type 2 DM (71 men and 77 nonpregnant women) signed informed consent and were entered into the study. The following inclusion and exclusion criteria were used. Patients who had a history of type 2 DM for more than one year were eligible for inclusion. Type 2 DM was diagnosed and classified according to clinical practice guidelines (twice fasting plasma glucose ≥7.0 mmol/L (126 mg/dL), 2-hour plasma glucose ≥11.1 mmol/L (200 mg/dL) during an oral glucose tolerance test, or random plasma glucose ≥11.1 mmol/L) [11]. Patients with acute kidney infection, renal tumors, impaired renal function (eGFR < 60 mL/min or proteinuria >30 *μ*g/mg), primary renal diseases, or secondary renal diseases were excluded. The collected data included gender, age, body height, and body weight. The eGFR was estimated by GFR (mL/min per 1.73 m^2^) = 186 × Pcr^−1.154^ × age^−0.203^ × 0.742 (if female) × 1.233 (if Chinese) [12]. All procedures followed were in accordance with the ethical standards of the responsible committee on human experimentation (Tri-service general hospital ethics committee, Taiwan, 097-05-201) and with the Helsinki Declaration of 1975, as revised in 2008.

### 2.5. Laboratory Measurement of Clinical Subjects

Plasma was separated from blood within one hour and stored at −80°C until analyzed. Plasma glucose levels were determined using the glucose oxidase method (YSI 203 Glucosed Analyzer; Scientific Division, Yellow Spring Instrument Company, Inc., Yellow Spring, OH). Total cholesterol (CHO) and triglycerides (TG) were measured using the dry, multilayer analytical slide method (Fuji Dri-Chem 3000 analyzer, Fuji Photo Film Corporation, Minato-Ku, Tokyo, Japan). The glycated hemoglobin A1c (HgbA1c) was evaluated by ion-exchange high pressure liquid chromatography method (Variant II; Bio-Rad, Hercules, CA).

### 2.6. Competitive Enzyme-Linked Immunosorbent Assay (ELISA)

According to* The ELISA Guidebook* (2001) edited by JR Croether, urine TCP-1*β* was determined using a competitive ELISA immunoassay in which TCP-1*β* (Abnova H00010576-M01, Taiwan) was coated with 100 *μ*L 1 *μ*g/well onto microtiter wells and the plate was incubated at RT overnight. Subsequently, 250 *μ*L 5% Skim Milk in PBST was added to the well at RT for 1 hour. The competition was achieved in 50 *μ*L human urine with the TCP-1*β* 50 *μ*L 1 *μ*g/mL at RT for 2 hours. Then, detection antibody by tag-specific 1 *μ*g/mL rabbit anti-GST antibodies (Abnova, PAB1625-E01P, Taiwan) was incubated at RT for 2 hours. HRP conjugated 1 : 40000 goat anti-rabbit IgG (Jackson, 111-035-003, PA, USA) was added for 1 hour. A chromogen TMB substrate (Biolegend, 421101, CA) solution was added to the wells to incubate for 30 mins at RT and then stopped by 2 N H_2_SO_4_. Finally, a microplate reader set to 450 nm was used to read the reaction.

For validation of our in-house ELISA, samples of high, medium, and low concentration in two replicates were used to calculate intra-assay and interassay coefficients of variation (CV). The intra-assay and interassay CV in our ELISA were 2% and 3%, respectively, which were within acceptable range.

### 2.7. Statistical Analysis

Analysis was performed using PASW Statistics version 18.0 statistical package for Windows (SPSS, Chicago, IL). In the mouse model, all experiments were repeated at least three times. Results within groups are expressed as the mean ± SE. Nonparametric analysis with Mann-Whitney *U* test was used to assess significant differences between two independent groups. In clinical investigation, one-way ANOVA using Bonferroni as a* post hoc* test was applied to compare differences among health, DM with hyperfiltration, and DM with nonhyperfiltration groups. Pearson product-moment correlation coefficient was used to assess the relationship between eGFR and urine TCP-1*β* level (1-OD) of competitive ELISA kit. All statistical data were expressed as two-sided, and *P* values less than 0.05 were considered to be statistically significant.

## 3. Results

### 3.1. Insulin Resistance and Change in *C*_cr_ among Control, HF, and Type 2 DM Mice

**Figure 1:**
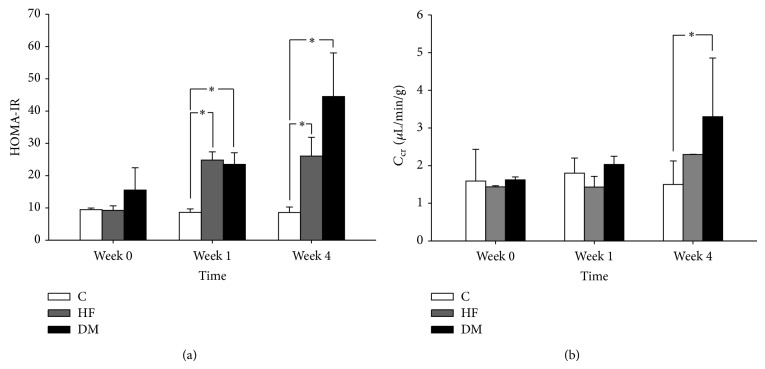
Change of insulin resistance (HOMA-IR) and creatinine clearance (*C*_cr_) in control (C), high-fat diet (HF), and type 2 diabetes mellitus (DM) mice groups. (a) The HOMA-IR levels in HF and DM groups were significantly higher than those in C group on week 1 and week 4 after induction. (b) The change of murine *C*_Cr_ in the DM group was significantly higher than in C group on week 4. ^∗^ *P* < 0.05.

Insulin resistance (HOMA-IR) is the hallmark of type 2 DM.  Figure 1(a) shows HOMA-IR gradually elevated in both HF and DM groups, suggesting that the typical characteristic of type 2 DM was successfully developed in our type 2 DM mice model. Compared to control and HF groups, the DM group developed insulin resistance sooner and a more prominent HOMA-IR elevation was obtained. Meanwhile, hyperglycemia, the fundamental characteristic of type 2 DM, is a defining criterion to judge the success of DM models. Together, hyperglycemia and insulin resistance, the two important clinical features of type 2 DM, could both be noted in our type 2 DM mice model.

Furthermore, our model was evaluated for its ability to mimic the hyperfiltration phase of early DN. All groups were further divided into 3 groups to represent the developing course in 0, 1, and 4 weeks after onset of type 2 DM.  Figure 1(b) shows that *C*_cr_ in the DM group had a mild increase at week 1 but a significant increase at week 4, demonstrating that this model had developed the hyperfiltration stage and mimicked clinical glomerular hyperfiltration of DN well. Thus, the first and fourth weeks after hyperglycemia were designated as representative hyperfiltration courses for the subsequent experiments.

### 3.2. Expression of TCP-1*β* in Renal Tissue and Urine Sample in Type 2 DM Mice

**Figure 2:**
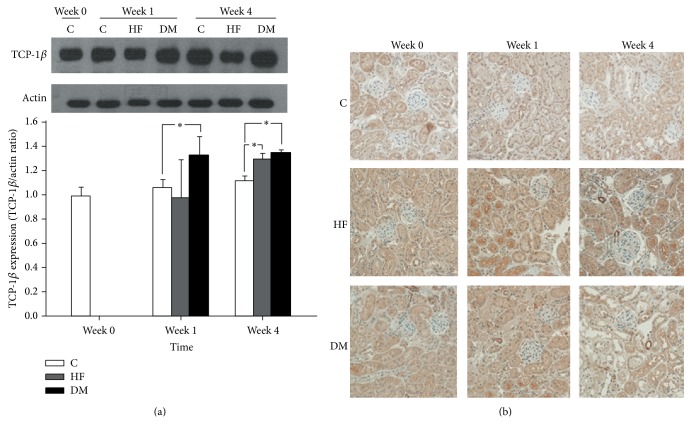
Expression of TCP-1*β* in renal tissue of mice. (a) As the representative Western blot shows, TCP-1*β* expression in the DM group was higher than in the C group at week 1 and week 4. In addition, TCP-1*β* expression in the HF group was higher than in C group at week 4. ^∗^ *P* < 0.05. (b) In IHC stain, as the representative pictures show, the expressions of TCP-1*β* in all groups were predominantly enhanced in the renal tubular region (original magnification, ×400).

After the success in developing a type 2 DM mice model, the expression of TCP-1*β* was investigated by kidney biopsy and WB. As Figure 2(a) shows, the amount of TCP-1*β* in renal tissue significantly increased in week 1 and week 4 in the DM group. As Figure 2(b) shows, an elevated TCP-1*β* level is expressed in the HF and DM groups in week 1 and week 4 and is located predominately on the renal tubules as compared with the glomerular region. While urine TCP-1*β* levels were too low to detect, the combined data (Figures 1 and 2) suggested that the highly expressed TCP-1*β* in renal tissues as evaluated by WB could be a possible biomarker for the diagnosis of hyperfiltration in early DN.

### 3.3. Expression of TCP-1*β* in Human Urine

To further explore the clinical evidence of TCP-1*β* associated with type 2 DM, 17 healthy subjects and 147 subjects with type 2 DM were enrolled in the study. Subjects with type 2 DM, classified as hyperfiltration (eGFR > 120 mL/min) and without hyperfiltration (eGFR < 120 mL/min), were grouped for studying the correlation with urine TCP-1*β*. The demographic data of these subjects is shown in Table 1. There was no significant difference between the two DM groups in HgbA1c and proteinuria. But the DM group with hyperfiltration was significantly younger and had lower serum creatinine. Mild hyperlipidemia with higher CHO and TG was noted in DM group with hyperfiltration.

**Figure 3:**
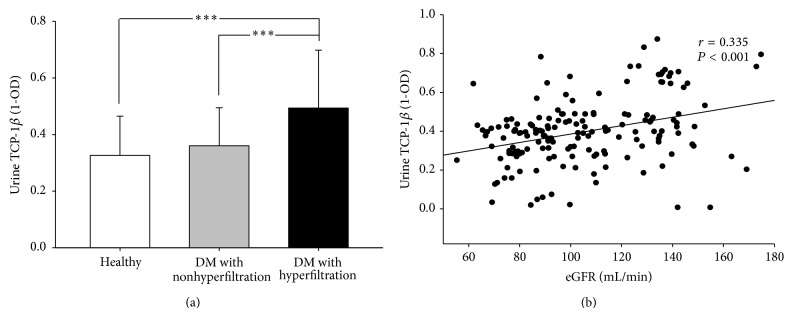
Expression of TCP-1*β* in urine sample in clinical subjects. (a) Urine TCP-1*β* levels (1-OD) in the group of DM with hyperfiltration were significantly higher as compared with healthy and DM with nonhyperfiltration groups. ^∗∗∗^ *P* < 0.001. (b) The urine TCP-1*β* (1-OD) level has a significantly positive association with eGFR in subjects with type 2 DM. (*r* = 0.335, *P* < 0.001).

In the clinical study, it was found that urine TCP-1*β* levels were significantly higher in the DM group with hyperfiltration (Figure 3(a)) and there was a weak correlation between the TCP-1*β* levels with eGFR (Figure 3(b); *r* = 0.335, *P* < 0.001). Thus, our clinical results could imply a possible role of TCP-1*β* in early DN.

## 4. Discussion

This study is an advanced extension of our previous* in vitro* mouse mesangial cell model and the first research identifying the role of TCP-1*β* in early DN in animal model and clinical subjects. In this study, we found that TCP-1*β* overexpressed in the tubules of kidney in type 2 DM mice with a simultaneous rise in *C*_cr_. In clinical investigation, we found urine TCP-1*β* levels were significantly higher in patients with type 2 DM in the hyperfiltration phase, in a linear association with eGFR levels.

The exact mechanism of hyperfiltration in DN is debated, with research supporting both glomerular and tubular theories of hyperfiltration in DN. Glomerular hyperfiltration and hypertrophy have been observed in the early stage of DN induced by STZ in mice [13]. TCP-1*β* is one molecular chaperonin, which is highly expressed during hyperglycemia-induced endoplasmic reticulum (ER) stress [14] and helps to fold not only the cytoskeletal-related proteins but also dozens of other proteins [15]. Our previous* in vitro* study found elevated TCP-1*β* levels in medium of murine mesangial cells treated with high glucose [5]. It was reasonably speculated that high glucose-induced ER stress on mesangial cells impedes the functions of cell migration and contraction, which influences the regulation of GFR. Consequently, TCP-1*β* on mesangial cells is upregulated for unfolding actin and tubulin. Other than mesangial glomerula, increasing resorption of glucose and sodium in proximal renal tubules also contributes to hyperfiltration in type 2 DM [16]. In addition, high glucose also induces renal tubular epithelial-mesenchymal transition, which may upregulate intracellular *α*-smooth muscle actins [17] and cause renal tubular fibrosis. However, our IHC stain showed TCP-1*β* predominantly expressed in renal tubular regions, instead of in mesangial glomerular region. We speculated that hyperglycemia-related hypocontraction of mesangial cells and progress of renal tubular fibrosis occurred simultaneously. Thus, TCP-1*β* was expressed in both mesangial cells and tubular cells. But expression of TCP-1*β* in renal tubules was so strong that TCP-1*β* in mesangial glomerula was obscured.

Wei et al. indicated that mice fed a HF diet showed elevated GFR and mesangial cells dedifferentiation, which increased expression of *α*-smooth muscle actin in glomeruli [18]. Severely obese subjects without type 2 DM also showed increased GFR and renal plasma flow by 51% and 30%, respectively, as compared with normal subjects [19]. Weight loss could significantly reduce GFR and renal plasma flow in obese subjects [20]. Accordingly, obesity-related renal injury, including glomerular hypertrophy and albuminuria, may contribute to DN [21]. Our mice fed a HF diet showed increased *C*_cr_ and insulin resistance as time went by, at higher levels than for the control mice, but lower than those for type 2 DM mice. However, expression of TCP-1*β* in renal tissue was elevated, similar to that of the type 2 DM mice. Accordingly, we may conclude that TCP-1*β* is more sensitive and appears before hyperfiltration develops.

The mechanism linking expression of TCP-1*β* and type 2 DM is still unclear. In a previous study with proteomic analysis, some subunits of TCP were overexpressed in skeletal muscle in obese subjects and patients with type 2 DM [22]. In our human clinical investigation, urine TCP-1*β* in DM with hyperfiltration was significantly higher in healthy subjects and DM with nonhyperfiltration. Juxtaposing these results with the results in our type 2 DM mouse model, our findings support the critical role of TCP-1*β* in early DN. Our study is the first to explore and identify urine TCP-1*β* levels for assessing patients with type 2 DM with hyperfiltration. These findings suggest that urine TCP-1*β* can be a useful and sensitive biomarker for detecting hyperfiltration in type 2 DM. Our method is easy and accessible for extensive clinical practice and can alert physicians to early DN in patients with type 2 DM and increased urine TCP-1*β*.

There are some limitations in our study. First, we used competitive ELISA for measuring urine TCP-1*β* in clinical subjects. In this method, antigen can interfere with the results, and sample on the test and bridge phenomenon cannot be avoided at extreme value [23]. Although we omitted the subjects with eGFR of less than 60 mL/min and proteinuria of more than 30 *μ*g/mg, various particles and metabolites in the urine may have caused false negatives and false positives. Furthermore, we did not measure the exact amount of urine TCP-1*β* quantitatively by competitive ELISA. Finally, we did not restrict the diets of subjects before the exam or investigate the treatment records of subjects with type 2 DM for other factors, such as hypertension or hyperlipidemia. Each subject's medications, food, and nutrition supplement may have influenced the competitive ELISA or TCP-1*β* in urine. However, overexpression of TCP-1*β* in hyperfiltration in the type 2 DM mouse model and clinical subjects could offset these limitations.

## 5. Conclusion

In summary, elevated TCP-1*β* in urine of patients with type 2 DM and in renal tissue of type 2 DM mice were both noted in the hyperfiltration stage. Hyperfiltration in DN may be detectable early by measuring urine TCP-1*β*, which can be a novel and valuable biomarker for clinical evaluation.

## Figures and Tables

**Table 1:** The general characteristics of subjects with healthy, type 2 diabetes mellitus (DM) with hyperfiltration and nonhyperfiltration groups.

	Healthy	DM with nonhyperfiltration	DM with hyperfiltration
*n*	17	98	49
Sex (male/female)	8/9	55/43	16/33
Age (year)	67.7 ± 12.8^2,3^	60.1 ± 12.0^1,3^	52.4 ± 9.3^1,2^
eGFR	89.3 ± 20.7^3^	91.3 ± 14.5^3^	138.7 ± 12.4^1,2^
FPG (mmo/L)	5.08 ± 0.65^2,3^	7.82 ± 2.36^1,3^	8.66 ± 2.64^1,2^
HgbA1c (%)	—	7.65 ± 1.63	8.23 ± 2.02
Creatinine (umol/L)	72.28 ± 13.71^3^	73.348 ± 14.36^3^	48.66 ± 8.01^1,2^
CHO (mmol/L)	5.26 ± 0.71^2^	4.51 ± 0.93^1,3^	4.84 ± 0.97^2^
TG (mmol/L)	1.12 ± 0.84^3^	1.49 ± 0.90	1.85 ± 1.34^1^
ACR (*μ*g/mg)	8.05 ± 5.50	11.14 ± 7.14	11.15 ± 5.71

eGFR: estimated glomerular filtrating rate; FPG: fasting plasma glucose; HgbA1c: glycated hemoglobin; CHO: total cholesterol; TG: triglyceride; ACR: ratio of urine microalbumin to creatinine; ^1^ *P* < 0.05 against health, ^2^ *P* < 0.05 against DM with nonhyperfiltration, and ^3^ *P* < 0.05 against hyperfiltration.
