# Corrigendum to “Chaperonin-Containing t-Complex Protein-1 Subunit *β* as a Possible Biomarker for the Phase of Glomerular Hyperfiltration of Diabetic Nephropathy”

**DOI:** 10.1155/2016/8653290

**Published:** 2016-03-16

**Authors:** Chung-Ze Wu, Li-Chien Chang, Yuh-Feng Lin, Yi-Jen Hung, Dee Pei, Jin-Shuen Chen

**Affiliations:** ^1^Graduate Institute of Clinical Medicine, College of Medicine, Taipei Medical University, Taipei, Taiwan; ^2^Division of Endocrinology and Metabolism, Department of Internal Medicine, Shuang Ho Hospital, Taipei Medical University, Taipei, Taiwan; ^3^School of Pharmacy, National Defense Medical Center, Taipei, Taiwan; ^4^Division of Nephrology, Department of Internal Medicine, Shuang Ho Hospital, Taipei Medical University, Taipei, Taiwan; ^5^Division of Endocrinology and Metabolism, Department of Internal Medicine, Tri-Service General Hospital, National Defense Medical Center, Taipei, Taiwan; ^6^Division of Endocrinology and Metabolism, Department of Internal Medicine, Cardinal Tien Hospital, Medical School, Catholic Fu Jen University, Xindian, Taiwan; ^7^Division of Nephrology, Department of Internal Medicine, Tri-Service General Hospital, National Defense Medical Center, No. 325, Section 2, Cheng-Kung Road, Neihu, Taipei 114, Taiwan

In the paper “Chaperonin-Containing t-Complex Protein-1 Subunit *β* as a Possible Biomarker for the Phase of Glomerular Hyperfiltration of Diabetic Nephropathy” [1] by C.-Z. Wu, L.-C. Chang, Y.-F. Lin, Y.-J. Hung, D. Pei, and J.-S. Chen, affiliation number 7 was incorrectly presented as “Division of Nephrology, Department of Internal Medicine, Tri-Service General Hospital, No. 325, Section 2, Cheng-Kung Road, Neihu, Taipei 114, Taiwan” and is corrected as above.

## References

[1] Wu C.-Z., Chang L.-C., Lin Y.-F., Hung Y.-J., Pei D., Chen J.-S. (2015). Chaperonin-containing t-complex protein-1 subunit *β* as a possible biomarker for the phase of glomerular hyperfiltration of diabetic nephropathy. *Disease Markers*.

